# Evaluation of wound healing, antioxidant and antimicrobial efficacy of *Jasminum auriculatum *Vahl. leaves

**Published:** 2016

**Authors:** Mittal Arun, Sardana Satish, Pandey Anima

**Affiliations:** 1 *Department of Pharmacognosy and Phytochemistry, Hindu College of Pharmacy, Sonipat, Haryana-131001, India*; 2 *Department of Pharmaceutical Sciences, Birla Institute of Technology,Mesra, Jharkhand-835215, India*

**Keywords:** *Jasminum auriculatum excision*, *Incision*, *Hydroxyproline content*, *Skin breaking strength*, *Antioxidant activity*, *Antimicrobial activity*

## Abstract

**Objective:**

To validate the ethno-therapeutic claim of the traditionally used plant *Jasminum auriculatum* (*J. auriculatum*) in skin diseases, by evaluating its wound healing potential along with its antioxidant and antimicrobial properties; so as to understand their role in wound healing.

**Materials and Methods:**

Excision and incision wound models were used to evaluate the wound healing activity on albino rats. The wound healing potential was assessed by measuring rate of wound contraction, epithelialization period, hydroxyproline content, skin breaking strength and histopathological parameters. Reference standard drug was Nitrofurazone ointment. The antioxidant activity was determined using 2, 2-diphenyl-1-picrylhydrazyl (DPPH) method. The antimicrobial activity was determined by agar well diffusion method and minimum inhibitory concentration by serial dilution method.

**Results:**

Higher rate of wound contraction (83.66±0.50% on 15th day), decrease in the period of epithelialization (17.83±1.6days), higher skin breaking strength (170.71±1.52g), higher collagen content and favourable histopathological changes revealed that topical application of ointment containing successive ethanolic extract (S.E.E) of *J. auriculatum* leaves has the most potent wound healing ability compared to control group in both the models studied. The DPPH radical scavenging activity of successive ethanolic extract was found to be 33.39µg/ml. Successive ethanolic extract was found to be most effective against *Pseudomonas auregenosa* having a zone of inhibition 16.65±0.6mm and the minimum inhibitory concentration was 0.78mg/ml.

**Conclusion:**

The data of this study indicate that successive ethanolic extract of the leaves exhibit potent wound healing, antioxidant and antimicrobial properties. This justifies the ethno-medicinal use of plant for the treatment of wound and microbial infections.

## Introduction

A wound is defined as the loss or breaking of the cellular and anatomical or functional continuity of the living tissue (Fulzele et al., 2002 ▶). Wound healing is the process of repair that follows injury to the skin and other soft tissues. Following injury, an inflammatory response occurs and the cells below the dermis begin to increase collagen production. Later, the epithelial tissue is regenerated (Souba and Wilmore, 1999 ▶). 

During natural healing process, an infection: mostly from *S. aureus, E. coli. P. auregenosa and Bacillus spp.* can delay it- by protracting the inflammatory phase, disrupting the normal clotting mechanism; hence, ultimately delaying angiogenesis (Mertz and Ovington, 1993 ▶).

Delaying inflammatory phase may also lead to generation of reactive oxygen species which, due to their damaging effects on cells and tissue, are noxious to wound healing process. The increase in free radical production and diminished antioxidant activity may worsen the condition and account for the delay in healing (Adly, 2010 ▶). 

Nowadays, a broad range of antibiotics are being used for management of wound infections, but these have been proved to have undesirable effects on the human body. Secondly, the pathogens have been successful in developing resistance against various commonly used antibiotics (Essawi and Srour, 2008 ▶).

In view of this, the plants have attracted attention, which have proven their use in herbal medicine and produc extracts containing biologically active compounds.

Plants and herbs with wound healing, antioxidant and antimicrobial potentials are being used in India, by customary healers; from time immemorial. However, few of them have been explored to confirm their potential healing effect but others still await the same. Hence, there is a need to introduce a scientific corroboration for the medicinal effect of these plants used in conventional medicine. So in order to evaluate and prove its claimed utility in folk medicine; *J. auriculatum* has been selected. Earlier, the extract of leaves of this plant in the form of various traditional formulations like jelly, juice or medicated ghee has been successfully applied on wounds (Despande et al., 1965 a, b ▶; 1966 a ▶, b ▶). 

Keeping in the view the claimed benefits of *J. auriculatum*, the present study was devised with special prominences to investigate the wound healing potential of successive extracts (i.e. petroleum ether, chloroform, ethanol and aqueous extracts) of *J. auriculatum* leaves by applying these topically. By measuring wound contraction, the most bioactive extract was identified, which was then evaluated in detail for wound healing, antioxidant and antimicrobial activities.

The acute dermal toxicity study of the extracts of leaves of the selected species was carried out to know its safety profile and for the selection of dose. 

## Materials and Methods

**Chemicals**

All the reagents and chemicals used were of analytical grade and purchased from Sigma Life Sciences and Hi-media, Mumbai, India.

**Plant collection and authentication**

Leaves were collected from the medicinal garden of Sri Venkateswara University, Tirupati (Andhra Pradesh). Herbarium so prepared was authenticated by Dr. H. B. Singh (Scientist F and Head, Raw Materials Herbarium and Museum, NISCAIR, New Delhi) under voucher specimen no. NISCAIR/RHMD/Consult/-2011-12/1763/63 and a specimen was deposited in the Department of Pharmacognosy, Hindu College of Pharmacy, Sonepat. 

**Preparation of extracts and qualitative chemical analysis**

Leaves were air dried in the shade and coarsely powdered. The extracts were prepared using 300 gm coarse powder of leaves by successive solvent extraction method using petroleum ether (60-80^o^C), chloroform, ethanol and purified water (ratio 1:8 of each solvent, soxhlation time 72 hrs each). The extracts obtained were concentrated using rota-evaporator. The concentrated extracts were then evaporated to dryness, in vacuum oven at temperature not more than 50^o^C. The dried extracts were stored at 2-8^o^C in refrigerator. All the extracts were tested qualitatively for the presence of various phytoconstituents, viz. alkaloids (Dragendroff’s test), carbohydrate (Molish test), flavonoids (using magnesium and dilute HCl), saponins (Frothing test), steroids and terpenoids (Liebermann-Burchard test) and tannins and phenolic compounds (5% ferric chloride test) (Sofowora, 1982 ▶; Trease and Evans, 1987 ▶). 

**Animals and microorganism**

The healthy albino rats of either sex, weighing 150-200g. were housed under standard environmental conditions of temperature and humidity (25±0.50C) and 12 h light/dark cycle. The animals were fed with standard pellet diet and water ad libitum. The animal studies were performed in the institute and experimental protocol was duly approved by Institutional Animal Ethical Committee (Reg No. 585/02/c/ CPCSEA India). Bacterial culture (*Bacillus subtilis, Staphylococcus aureus, Pseudomonas aureginosae, Micrococcus luteus and Escherichia coli*) and fungal culture (*Candida albicans and Aspergillus niger*) were procured from Microbial Type Culture Collection, Chandigarh, India.

**Acute dermal toxicity**

The limit test for the acute dermal toxicity study was designed as per OECD guidelines no.434 and carried out in adult female albino rats by fixed dose method. Each animal was weighed and its body surface area calculated. Body surface area equal to 10% of total was properly shaved and extracts were incorporated in water soluble ointment base prepared by using PEG 4000 and PEG 400, applied topically (2000mg/kg body weight) to the shaved area (Allen et al., 2008 ▶; Yaduvanshi et al., 2011 ▶). The ointment was restricted to the shaved area using cotton gauge and a non-irritating tape. Animals were observed continuously for 24 hrs, for any sign of toxicity and then periodically for the next 13 days.

**Wound healing activity**

*Preliminary wound healing screening of the extracts*

Excision wound model was used and wound contraction measured. In this way the most bioactive extract was identified. One tenth of the maximum tested dose was applied using the ointment having 16% concentration of the extract in ointment base (Nalwaya et al., 2009 ▶). The animals were divided into five groups of six animals each.

Group I: served as control treated with ointment base.

Group II, III, IV, V: received the application of successive petroleum ether, chloroform, ethanolic and aqueous extract ointments.

All the animals in each group were anaesthetized by open mask method using anaesthetic ether. The rats were depilated at dorsal thoracic region and full thickness of skin (500mm2) was cut off from a pre-determined area on the dorsal back of rats 1cm away from vertebral column and 5cm away from the ear. The wound area was measured immediately by tracing it on transparent paper and calculated using 1mm graph sheet. (Morton and Malone, 1972 ▶; Muthusamy et al., 2008 ▶).

For measuring wound contraction, the same protocol was followed every 3^rd^ day till complete healing. The percentage of wound contraction area was calculated. The area of wound at the time of wounding was considered as 100% and the wounding day was considered as day zero. 

**Detailed wound healing activity**

The detailed wound healing activity of the most bioactive extract was evaluated by monitoring various parameters of excision and incision wound models.

The animals in both the models were divided into three groups of six animals each.

Group I: Control group, received ointment base.

Group II: Standard group, treated with 0.2%w/w Nitrofurazone ointment.

Group III: Test group, treated with successive ethanolic extract ointment. 

The treatment was given topically once a day, starting from the wound induction till complete healing.

**Excision wound model**

The excision wound model was performed according to the method as described above. The various parameters such as wound contraction percentage, epithelialization period, hydroxyproline content and histopathology of granular tissues were evaluated (Werner et al., 1994 ▶; Neuman and Logman, 1950 ▶).

**Incision wound model**

The animals were grouped as described in excision model. The animals were anaesthetized using ether anesthesia. One full thickness paravertebral incision of 6cm length was made through the skin, on either side of the vertebral column with the help of sterile scalpel (Ehrlich and Hunt, 1969 ▶).

After complete haemostasis, the parted skin was kept together and stitched with black silk surgical thread (no. 000) and curved needle (no. 11) at 0.5cm intervals. The continuous threads on both wound edges were tightened for better closure of the wounds. The wounds of animals in different groups were treated with topical application of the ointment as described above for the period of 10 days. The wounding day was considered as day zero. 

After thorough healing of wounds, the sutures were removed on the 8th post wounding day and wound breaking strength was determined on 10th day by continuous constant water flow technique (Lee 1968 ▶). The regenerated tissues from the healed lesions of wound were used for estimation of hydroxyproline content (Neuman and Logan 1950 ▶).

**Histopathology**

For histopathological examination; samples of healed skin tissue were taken from the animals of control, standard and treated groups of excision and incision wound models (Shenoy et al., 2011 ▶).

**In-vitro activities of bio active extract**

*Antioxidant activity*

The free radical-scavenging activity was measured in terms of hydrogen donating or radical-scavenging ability using stable radical DPPH (2, 2-diphenyl-1-picrylhydrazyl) and total phenolic content was determined by the Folin-Ciocalteu method (Djeridane et al., 2006 ▶; Anna et al., 2012 ▶). The results are expressed in IC50 (inhibitory concentration 50) values and mg gallic acid equivalents per gram of dry mass of extract (mg GAE/g).

*Antimicrobial activity*

Agar well diffusion technique was used to evaluate antimicrobial activity. The extract was dissolved in DMSO (dimethyl sulphoxide) and used at a concentration of 50 mg/ml. The minimum inhibitory concentration (MIC) was also determined by serial dilution method. The concentrations of the extract used were 25.0, 12.5, 6.25, 3.125, 1.562, 0.781 and 0.39 mg/ml. Ciprofloxacin and fluconazole (100 μg/ml) were used as standard antibacterial and antifungal agents, respectively (Cappucino and Sherman, 1996 ▶; Vats et al., 2011 ▶). The agar well treated as control contains only DMSO. 

**Statistical analysis**

The mean value ± SEM was calculated for each parameter. Results were statistically analyzed by one-way-analysis of variance (ANOVA) followed by Dunnet’s t-test. P< 0.05 was considered as significant.

## Results

**Qualitative chemical analysis**

The preliminary phytochemical screening revealed the presence of alkaloids, carbohydrates, flavonoids, steroids, terpenoids, saponins, tannins and phenolic compounds. 

**Acute toxicity study**

The extracts of leaves were found to be safe upto 2000 mg/kg of body weight. Even after 24 hrs of dermal application of extracts, there was no sign of toxicity; hence, the extracts were considered to be safe. So one tenth of the maximum tested dose (200mg/kg) was selected for investigation.

**Wound healing activity**

*Preliminary wound healing screening*

**Figure1:**
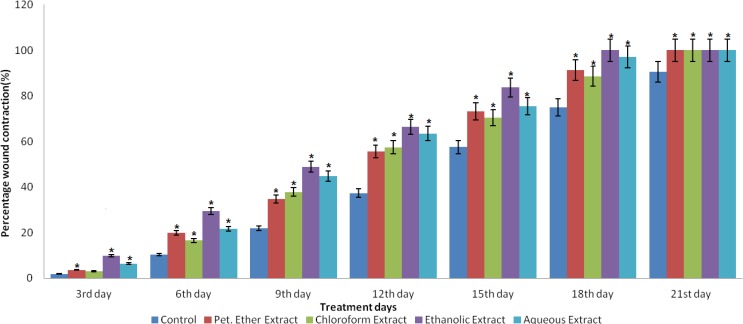
Effect of different extracts of *J. auriculatum* on excision wound expressed as percentage of wound contraction.Values are represented as mean± SEM (n=6). Data wereanalyzed by one-way Anova followed by Dunnett’s test *p<0.05 as compared to control

The preliminary wound healing screening of all the extracts revealed that the successive ethanolic extract (S.E.E) has the best wound healing potential compared to the other extracts (Figure 1). So, for the further detailed wound healing activity, this extract was selected. 

*Detailed wound healing activity*

In both the studied models, significantly improved wound-healing activity has been observed with successive ethanolic leaves extract of *J. auriculatum*. 

In excision wound model, successive ethanolic extract treated animals showed significant reduction in wound area (P< 0.05), faster rate of epithelialization (17.83±1.6 days and increased hydroxyproline content (P< 0.05) when compared with the control group of animals. Table 1 shows the wound contraction percentage and other biochemical observations of all the three groups of animals in excision wound model. 

**Table 1:** Effects of topical application of *J. auriculatum *in excision wound model

**Groups**	**% Wound contraction**						**Period of epithelialization (days)**	**Hydroxyproline** **Content** **(µg/mg)**
	**Day 3**	**Day 6**	**Day 9**	**Day12**	**Day15**	**Day 18**		
**Control**	1.90±0.19	10.42±0.38	21.94±0.73	37.32±0.55	57.53±0.87	74.96±0.81	22.83±0.40	79.48±0.95
**Std**	15.04±0.8*	36.69±0.85*	57.03±1.14*	72.93±1.50*	94.48±1.61*	100.00±0.00*	16.33±0.21*	145.4±1.02*
**S. E. E**	9.77±0.61*	29.41±0.51*	48.89±0.76*	66.39±0.94*	83.66±0.50*	100.00±0.00*	17.83±0.16*	84.0±1.65*

Values are represented as mean± SEM (n=6). Data were analyzed by one-way Anova followed by Dunnett’s test

S. E. E= Successive ethanolic extract.

* p<0.05 as compared to control. Std= Nitrofurazone;

Table 2 depicts the wound healing effects of *J. auriculatum* in incision wound model. In incision wound model, successive ethanolic extract treated animals demonstrated significant skin breaking strength up to 170.7± 1.52 when compared to control animals (144.7±0.25). Significant increase in hydroxyproline (P< 0.05) content was observed in animals treated with successive ethanolic extract compared to the control group of animals.

**Table2:** Effects of topical application of *J. auriculatum*in incision wound model

**Parameter**	**Control**	**Std**	**S. E. E**
**Skin Breaking strength(g)**	144.7±0.25	195.0±1.46*	170.71±1.52*
**Hydroxyproline content(µg/mg)**	61.37±0.74	138.30±1.07*	64.05±0.92*

Values are represented as mean± SEM (n=6). Data wereanalyzed by one-way Anova followed by Dunnett’s test

* p<0.05 as compared to control. Std= Nitrofurazone; S. E. E= Successive ethanolic extract.

**Histopathology analysis**

**Figure 2(a-f):**
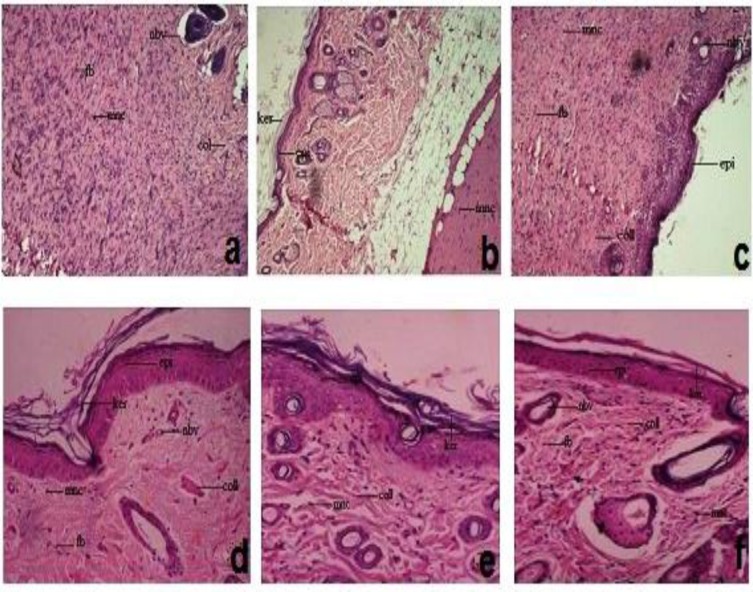
Histopathology of skin in standard, successive ethanolic extract and control treated groups. Skin histopathology of standard(a, d), successive ethanolic extract treated group (b, e) and control group (c, f) in excision and incision wound models respectively. fb: Fibroblast cell; mnc: Mononucleated cell; epi: Epithelilization; ker:

In Figure 2, in both models, treatment of animals with standard drug and successive ethanolic extract of *J. auriculatum* showed significant healing as in fibroblasts cells, collagen fibers and new blood vesicles (Figure A-D) while in control rats wounds showed incomplete healing (Figures E and F). The control group showed the slightest wound healing ability when compared to extracts and reference ointment treated groups. Fibroblast cells, collagen fibers and new blood vessels are prominently present in standard and extract treated groups as compared to control. 

**Antioxidant activity**

Total phenolic content of successive ethanolic extract of *J. auriculatum* was 8.47mg/g weight of the dry extract and IC50 values obtained were 33.39µg/ml for successive ethanolic extract and 35.41µg/ml for ascorbic acid as given in Table 3.

**Table 3:** *In-vitro* antioxidant activity of *J. auriculatum*

**Sample**	**Total Phenolic content** **(mg GAE/gm)**	**DPPH radical scavenging activity** **(IC**_50 _ **value µg/ml)**
**S. E. E (** ***J. auriculatum*** **)**	8.47±1.34	33.39±0.74
**Standard ** **(Ascorbic acid)**	-------	35.41±0.66

All values are expressed as mean± SEM of three replicate experiments; S. E. E= Successive ethanolic extract, GAE=Gallic acid equivalent, IC_50_= Inhibitory concentration50, DPPH= 2, 2-diphenyl-1-picrylhydrazyl

**Antimicrobial activity**

The successive ethanolic extract has shown inhibition effect on the growth of all the organisms tested. But its efficiency in inhibition varies for each organism. *J. auriculatum* successive ethanolic extract has shown inhibition range of 11-16 mm. All IZDs (Inhibition Zone Diameter) corresponding to the test organism are tabulated in Table no.4. Ciprofloxacin and Fluconazole have shown IZD ranges from 15-24 mm and16-17mm at a concentration of 100 µg/ml. The MIC value of extract is shown in Table no. 5. *Pseudomonas aeruginosa* was found to be the most sensitive microbe with the MIC value of 0.78 mg/ml whereas MIC values of fluconazole for both fungi were found to be 2.5 μg/ml. Ciprofloxacin exhibited MIC 1.25 μg/ml against *Pseudomonas aeruginosa* and 2.5 μg/ml against all the other bacteria.

**Table 4.** Zone of inhibition of successive ethanolic extract of *J.auriculatum* on experimented microbes

**Micro-organism**	**Zone of Inhibition in mm**	
	**Test Drug(mg/ml)**	**Standard Drug(µg/ml)**
***Bacillus subtilis***	13.50±1.4**	16.96±1.8
***Staphyloccocus aureus***	12.42±1.1*	15.67±2.22
***Pseudomonas aeruginosa***	16.65±0.8**	24.33±1.12
***Micrococcus luteus***	11.45±1.3*	17.21±2.49
***Escherichia coli***	11.35±0.6*	19.67±2.11
***Candida albicans***	----	16.77±1.11
***Aspergillius niger***	----	17.43±1.19

Values are expressed as mean±SD of three replicate experiments. (Ciprofloxacin and Fluconazole are the standards for bacteria and fungus, respectively). DMSO did not show any inhibitory potential.

* P<0.05,

** P<0.01

**Table 5:** Minimum inhibitory concentration effects of successive ethanolic extract of *J.auriculatum* on experimented microbes

**Micro-organism**	**Minimum inhibitory concentration**	
	**Test Drug(mg/ml)**	**Standard Drug(µg/ml)**
***Bacillus subtilis***	1.56	2.5
***Staphyloccocus aureus***	6.25	2.5
***Pseudomonas aeruginosa***	0.78	1.25
***Micrococcus luteus***	3.125	2.5
***Escherichia coli***	12.5	2.5
***Candida albicans***	-----	2.5
***Aspergillius niger***	-----	2.5

## Discussion

Wound healing is a sequence of events which consists of coagulation, inflammation, collagenation, wound contraction and epithelialization (Murti and Kumar, 2012 ▶; Mittal et al., 2013 ▶).While the phase between coagulation to collagenation is intimately inter-linked, the phase of wound contraction and epithelialization are independent to each other and run concurrently (Bairy and Rao, 2001 ▶; Shirwaikar et al., 2003 ▶). 

In order to evaluate wound healing activity, no single model is adequate to collectively represent the various components of the wound healing process as a whole. Hence, in the present study two different wound models were used to establish the healing potential of successive ethanolic extract of *J. auriculatum* on various phases.

Collagen, the major protein of extracellular matrix, is the component which gives strength, support and integrity to the wound matrix. Breakdown of collagen liberates free hydroxyproline and its peptides. A healing tissue synthesizes collagen, which is a constituent of growing cells. Therefore, measurement of hydroxyproline can be used as an index for collagen turnover (Nayak et al., 2006 ▶; Gurung and Basnet, 2009 ▶).

The results obtained in the present study proved that all successive extracts i.e. petroleum ether, chloroform, ethanol and aqueous have wound healing efficacy when evaluated by measuring wound contraction using excision wound model. The ethanolic extract shows comparatively better wound healing potential and hence, was selected for detailed study. The increased rate of wound contraction, enhanced epithelialization and increased hydroxyproline content lead to faster healing as confirmed by the increased healed area in the successive ethanolic extract results compared to the control ones. This enhancement may be due tothe enhanced collagen synthesis (Jayasutha et al., 2011 ▶).

It was also found that successive ethanolic extract treated incised wound exhibited an increased skin breaking strength compared to control. This means increased collagen concentration and stabilization of fibers (Suruse et al., 2011 ▶).

The results shown above are further supported by histopathological evidences i.e. enhanced Keratinization, epithelialization, collagenization and neovascularisation in the extract treated wounds compared to control. Thus, the successive ethanolic extract of leaves promotes wound healing by increased cell proliferation and collagen deposition. 

As per the literature survey, the presence of reactive oxygen species and microbes at the wound site has synergetic effects causing delay in healing. The treatment of such infected wounds is a big challenge. From the results it is clear that successive ethanolic extract of leaves possesses good antimicrobial and antioxidant effects. 

The preliminary phytochemical analysis revealed the presence of flavonoids, triterpenoids, steroids, alkaloids, saponins, tannins and phenolic compounds in successive ethanolic extract. As these agents influence one or more phases of healing process, hence, accelerating it.Flavonoids are known to reduce lipid peroxidation not only by preventing or slowing the onset of cell necrosis but also by improving vascularity. Hence, any drug that inhibits lipid peroxidation is believed to increase the viability of collagen fibrils by increasing the circulation, preventing the cell damage and by promoting the DNA syhthesis (Getie et al., 2002 ▶).

Flavonoids and Triterpenoids are also known to promote the wound-healing process mainly due to their anti-microbial and free radical scavenging, which seems to be responsible for wound contraction and increased rate of epithelialization (Tsuchiya et al., 1996 ▶; Scortichini and Pia, 1991 ▶).

Thus, wound healing property of *J. auriculatum* may be attributed to the phytoconstituents present in it, which may be either due to their individual or additive effect that hastens the process of wound healing. From the present study, it is concluded that all of the successive extracts i.e. petroleum ether, chloroform, ethanol and aqueous extracts possess wound healing potential. Measurement of wound contraction in excision wound model concludes that ethanolic extract is more potent. Detailed study upon this extract based on measurement of wound contraction, lesser epithelialization period, increased tensile strength, increased collagenation, histopathology, antioxidant, antimicrobial and phytochemical analysis supports the idea that successive ethanolic extract has remarkable effects. Further studies with purified constituents are needed to understand the complete mechanism of wound healing potential and constituents responsible for the same activity*.*

## Conflict of interests

There is no conflict of interests.
